# Interactions between vulvovaginal disorders and urinary disorders: The case for an integrated view of the pelvis

**DOI:** 10.1016/j.ijwd.2021.09.006

**Published:** 2021-09-24

**Authors:** Gayle Fischer, Jennifer Bradford

**Affiliations:** a Department of Dermatology, Faculty of Medicine, University of Sydney, Sydney, New South Wales, Australia; b Department of Obstetrics and Gynaecology, Blacktown Hospital, Sydney, New South Wales, Australia

**Keywords:** Bladder, Urethra, Vulvovaginal skin, Vulvodynia, Dyspareunia

## Abstract

Lower urinary tract symptomatology is often difficult to categorize if history and investigation focus only on the urinary tract. Disease and dysfunction in organs more posteriorly can often cause or influence such bladder and urethral symptoms. Vulvovaginal skin diseases are an important but often missed influence on lower urinary tract symptomatology.

 

**What is known about this subject in regard to women and their families?**

• Vulval disease has a profound effect on quality of life, including sexuality, emotions, and activities of daily living (Wijaya et al., 2021).
• The vulval quality of life index is an instrument that measures the impact of vulval dermatoses (Saunderson et al., 2020).
• Effective treatment improves quality of life, and the vulval quality of life index may be used to monitor treatment response.

**What is new from this article as messages for women and their families?**

• The majority of vulval diseases are skin conditions that respond to appropriate dermatological treatment.
• Urinary incontinence has a profound effect on exacerbating symptoms in vulval disease.
• Bladder dysfunction may be caused or exacerbated by disease or dysfunction more posteriorly in the pelvis.

## Introduction

Pelvic symptomatology is often multifactorial in etiology due to the close proximity of the pelvic viscera to each other, the pelvic floor, and the bony pelvic girdle (which also encompasses the lower spine and hip joints). Thus, a patient who presents with disease or dysfunction in one viscerum may have substantial symptomatic sensory input from other pelvic problems or even spinal and hip dysfunction. There are two generic mechanisms whereby different components of the pelvis may interact: emissions from the bladder, vagina, and bowel, contributing to either infection or various dermatoses, and neuromuscular interactions promoted by the pelvic floor complex and bony pelvis. This review concentrates on vulvovaginal disorders and how they interact with urinary disorders.

## Anatomy

In additional to genital epithelium, the functional anatomy of the lower pelvis contains five components, arranged posterior to anterior: 1) lumbo-sacral spine; 2) sigmoid colon, rectum, and anus; 3) vulva and vagina; 4) bladder and urethra; and 5) the pubic symphysis, where the two innominate bones join. All of these elements are joined by the pelvic floor: a complex myofascial structure that provides support and sphincter function to the viscera, but also allows for referral of pain and dysfunction from one part to another.

In general, referral of pain and dysfunction in the lower pelvis tends to be posterior to anterior. This means that the bladder and urethra, the most anterior parts of the pelvis, are especially susceptible to influence from disease or dysfunction in other parts. It is well known that colorectal disorders influence bladder function, but vulvovaginal disorders are also capable of the same influence. Since the lumbosacral spine forms the posterior portion of the pelvic girdle, it is also unsurprising that spinal disorders can also influence bladder function, not to mention the lower bowel and vulva and vagina.

## Etiology of vulval discomfort

In our published experience with vulval discomfort, we have found that approximately two thirds of cases are caused primarily by genital dermatoses and approximately one third by neuromuscular disorders occur inside or outside of the pelvis. Only 4% of cases were not able to be characterized (Harris et al., 2017). About one quarter of cases are multifactorial. Therefore, if a patient presents with pelvic symptoms that do not adequately improve after successful treatment of the presumed etiological event, a secondary cause for the symptoms should be sought in more posterior organs.

We contend that the rate of primary psychological causes for vulvovaginal discomfort is no higher than that for any other physical presentation in medicine. This includes the presentation of dyspareunia. It is of some concern to us that nonmedical sex therapists see primary presentations of female dyspareunia that have not been examined by a doctor.

## Dermatoses

The most common dermatoses arising on genital skin are dermatitis (56%), chronic vulvovaginal candidiasis (CVVC; 10%), psoriasis (PS; 7%), and lichen sclerosus (LS; 4%; Fisher and Bradford, 2016). These are all chronic diseases that require long-term management. On the other hand, genital dermatoses are heavily influenced by urinary frequency and incontinence (as well as urinary incontinence products), menstruation and menstrual products, defecatory disorders (especially diarrhea, frequency, and incontinence), activities of daily living and exercise/sports that cause sweating, and clothes (especially stretch and occlusive).

Secret women’s business (i.e., regular personal hygiene practices that women engage in) is also an important consideration. In an audit of 500 consecutive vulvovaginal presentations, 75% of patients did not improve until their unhelpful habits were identified and stopped, including clothes (26%), pads/liners (9%), topical antifungals (8.5%), and soaps/cleansers (8%; Fisher and Bradford, 2016). Therefore, genital dermatoses in women may not be adequately managed if the dermatological issues alone are addressed. It is essential to be able to identify and manage a range of other pelvic problems for which many clinicians do not have appropriate training.

## Neuromuscular disorders

Approximately one third of all genital presentations have input from spinal, hip, or lower limb disease or dysfunction (Coady et al., 2015; Gyan et al., 2013; Harris et al., 2017). Although dysfunction in the pudendal nerve is often invoked for vulval pain, it is important to realize that more general lumbosacral disease and dysfunction may more commonly produce identical patterns of pain. The pelvic floor functionally acts as both support and sphincter for the pelvic viscera. This is true not only for the bladder, rectum, and anus, but also for the vagina. An apparent reduction in vaginal introital dimensions may be caused not only by scarring dermatoses, such as LS, but also by localized pelvic floor spasm. Although this spasm may be caused by dermatoses of the overlying skin, it may be primarily or secondarily caused by bowel disorders, spinal problems, or hip dysfunction.

## Presentation

Vulval disorders usually present as localized vulvovaginal symptomatology, but many presentations are not of the primary etiology but rather secondary symptoms. Much like urinary dysfunction and pain, vulvovaginal disorders may present with sexual and relationship difficulties. For example, a retrospective audit of 166 consecutive presentations of chronic, intractable vaginal dyspareunia revealed that 61% were caused by dermatological diseases and that 88% of these were chronic vulvovaginal candidiasis (33%), lichen planus (21%), lichen sclerosus (19%), and PS (15%; Fischer and Bradford, 2016).

Patients tend to tailor their histories to that of the specialty of the clinician they are seeing, and intake patient questionnaires may be similarly skewed. Thus, it is not uncommon for us to see women who have presented to a urologist with apparently idiopathic bladder dysfunction but have not revealed their coexisting vulval symptoms, which may turn out to be etiological.

## Clinical approach to vulval presentations

History-taking should include inquiry for symptoms in the following categories: urinary, vulvovaginal, anorectal, and spine and hip (including operation and trauma). Examinations should be done with a high degree of suspicion for a dermatological entity. A low vaginal culture should always be taken, with the caution that antifungals in the previous month may render the result inconclusive. Furthermore, in a healthy nonpregnant woman, a pure vaginal isolate of group B streptococcus is usually not clinically relevant. If there is genuinely no skin abnormality and the vaginal culture results are normal, then a primary musculoskeletal etiology should be considered.

### Contact dermatitis

Dermatitis may be endogenous (usually atopic) or exogenous in etiology. On the vulva, exogenous contact dermatitis is the most common dermatosis, more common than endogenous dermatitis, although these patients are often atopic and therefore more prone to react to irritants. Contact dermatitis is caused by external agents, such as irritants (common) and allergens (uncommon), and is potentially curable if the triggers are identified and ceased and the rash is treated.

Contact dermatitis is predominately itchy, but there may be burning if the mucosal surface is involved. Excoriations from scratching and fissures produce pain and dyspareunia. The symptoms are normally worse at night and interfere with sleep. Heat, sweat, and friction (including intercourse) often exacerbate dermatitis. Examination shows poorly defined erythema of the labia, vaginal vestibule, and perineum, which may extend onto the mons pubis, inner thighs, and perianal skin. In long-standing cases, lichenification (i.e., thickening of the skin) is common, resulting in rugosity, scale, and pallor of the labia majora and perianal skin. The vagina is not involved; however, patients may report an offensive discharge because of desquamation from the introital skin. Superinfection identified on culture must be simultaneously treated because this is an antigenic trigger.

In all patients with dermatitis, urinary and fecal incontinence (and therefore pad wearing) will exacerbate the problem. Wetness increases the likelihood of irritation and formation of allergic reactions. Seminal fluid often exacerbates an already established dermatitis.

Treatment of all forms of dermatitis includes topical corticosteroid ointments. For contact dermatitis, treatment involves the identification and cessation of the offending trigger and 1% hydrocortisone ointment applied twice daily to the affected skin, usually for 4 weeks.

### Irritant contact dermatitis

Irritant vulval dermatitis presents with a red rash in the area in contact with the irritant, usually only the labia majora and perineum. Common causes include

• chemicals in feminine hygiene products, cleansers and imidazole products,
• occlusive stretch clothing, pads, and liners,
• incontinence of urine and feces,
• waxing and shaving, and
• intense sports and exercise, especially cycling.

### Allergic contact dermatitis

Allergic contact dermatitis is a type IV response to an allergen and is fortunately uncommon. The most frequent causes include latex, imidazole creams (which may also be irritants), estrogen creams, fragrance, diphenhydramine, antibiotics, topical anesthetics (particularly benzocaine), wet wipes containing preservatives, and topical amitriptyline. Rarely, seminal fluid may cause a true contact allergic reaction.

### Psoriasis

PS is the most common endogenous skin condition chronically involving the vulva (Kapila et al., 2012). It occurs in up to 5% of the adult population, and genital skin is commonly affected. Most patients with genital PS have a positive personal or family history of PS. However, not all patients are aware of their history, and PS may be diagnosed for the first time when skin signs external to the vulva, such as plaques on the elbows and knees, are identified. Historically, PS differs from dermatitis by its episodic nature, often triggered by stress, illness, or the premenstrual phase. A common history is chronic episodic itchy vulvitis, which responds temporarily to topical corticosteroids. Genital PS lacks the scale and sometimes sharp edge of plaque PS. When scale occurs in vulval PS, it is usually in the interlabial sulcus. Other folds affected include the gluteal cleft, inguinal crease, and inframammary, as well as the umbilicus.

The lesions are usually more erythematous and well defined than dermatitis and are usually but not always bilaterally symmetrical. The rash can extend inward as far as the vestibule. The perianal area and natal cleft may be involved. Natal cleft involvement is a useful sign because this is not seen in dermatitis. Lichenification of the perianal skin and labia majora may be severe. A culture taken from the lower vagina should be tested to exclude candidiasis, with which PS is often associated. PS is a clinical diagnosis, and biopsy is usually unhelpful in the genital area where, unlike other parts of the skin, it tends to be nonspecific. Treatment guidelines have been detailed elsewhere (Fischer and Bradford, 2016).

### Case history

A 47-year-old premenopausal woman presented with entry dyspareunia and urinary frequency 6 months after an episode of apparent fungal vulvovaginitis. Her urine and vaginal cultures were normal. She was seen by a gynecologist, and the prescribed topical estrogen and antifungals made symptoms worse with standing. The patient was also seen by a urologist, and cystoscopy and urodynamics were unremarkable. Finally, she was seen by a pelvic physiotherapist, and muscular trigger-point therapy was unhelpful.

After many years of proctalgia, the patient indicated that symptoms were worse with standing and defecation and improved when lying down. She was referred to a colorectal surgeon, but sphincterotomy was unhelpful. Chronic low-back pain commenced at the time of the anal pain.

**Figure 1:**
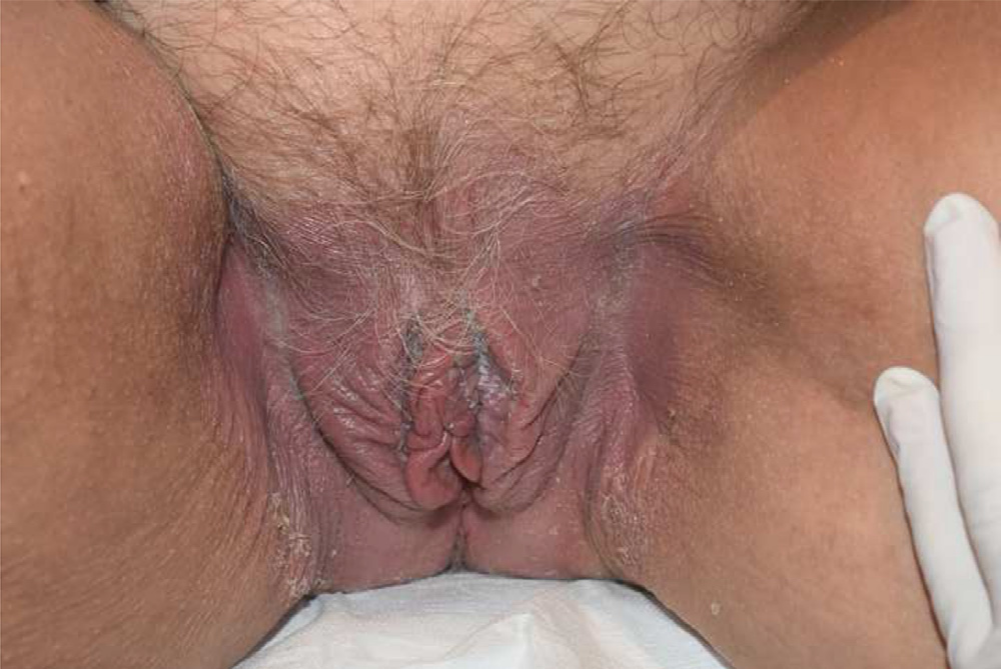

Examination showed a well-demarcated symmetrical and bilateral erythematous vulval rash extending from the vulval hairline into the vaginal introitus, extending out to both groins (Fig. 1). Typical psoriatic plaques were noted on both knees and the scalp hairline.

Initial management included reducing heat, sweat, and friction; moderate-potency corticosteroid ointment (methyl prednisolone aceponate 0.1%) nocte, and 2% liquor picis carbonatum ointment mane (coal tar derivative) after showering mane.

At 6 weeks of treatment, urinary frequency and entry dyspareunia reduced, but the anal pain remained unchanged. Upon examination, the PS was shown to be objectively controlled. Pubococcygeus spasm was noted, palpation of which reproduced dyspareunia. In addition, anal spasm was noted, palpation of which reproduced anal pain. Subsequently, treatment with corticosteroid was ceased, 2% liquor picis carbonatum ointment was continued twice daily. The patient was referred to a physiotherapist with pelvic and spinal skill sets. The treatment outcome was a gradual but complete resolution of dyspareunia, urinary frequency, anal pain, and low-back pain.

### Comment

This patient’s presenting symptoms were secondary to an underlying dermatosis. This was not comprehended, so she was initially referred to a urologist. Initial treatment was of the primary problem, and only when this had been addressed was she able to benefit from pelvic floor physiotherapy to address muscle spasm, which caused secondary urinary and spasm-induced symptoms.

## Chronic vulvovaginal candidiasis

In healthy women, CVVC occurs only with adequate estrogen levels (Fischer and Bradford, 2011; Hong et al., 2014). Therefore, it occurs only in menstruating women or with estrogen replacement in postmenopausal women. Acute and recurrent candidiasis are caused by infection with *Candida* organisms, almost always *albicans*. These episodes are well defined and adequately treated with over-the-counter antifungals, and the patient is asymptomatic in between episodes.

CVVC is very different, however. The pathogenesis is unknown, but CVVC behaves not as an infection but as a local maladaptive response of the genital epithelium to the commensal candida organism, which effectively becomes an antigen. CVVC is also almost always linked to *C. albicans*; however, non-*albicans* isolates (e.g., *C. glabrata*) may be implicated, particularly in diabetic and immunocompromised individuals. Unlike acute candidiasis, symptoms usually remit during pregnancy. Combined oral contraceptive pills are hardly ever implicated.

CVVC presents with recurrent or constant itch, dyspareunia, swelling, and pain. These symptoms tend to cycle, being worse premenstrually and improving on the first day of menstruation. Unlike acute candidiasis, discharge does not always occur. Antibiotics and topical corticosteroids typically exacerbate or precipitate symptoms. Short courses of antifungals often improve symptoms temporarily. Frequent use of over-the-counter antifungals explains why vaginal culture tests are often negative. Therefore, a negative vaginal culture does not rule out this condition, particularly if other aspects of the history are present. The patient’s partner may sometimes experience postcoital penile itch, which is characteristic.

Typical examination is of a vulvovaginitis with erythema of the labia minora, extending into the sulcus between the minora and majora. Sometimes the examination is completely normal. A culture taken from the lower vagina should be tested, but biopsy is unhelpful, showing only nonspecific changes seen in dermatitis.

Chronic vulvovaginal candidiasis can be a very difficult diagnosis. If the history is typical but examination and cultures are normal, we recommend a trial of treatment with prolonged antifungal therapy. Such a trial often becomes the ultimate diagnostic test. We recommend fluconazole 50 to 100 mg daily until symptoms subside, then tapering the dose over the next few months, depending on the clinical response. In menopausal women on estrogen replacement therapy, CVVC is usually controllable within 1 month of oral azole treatment if all estrogen is stopped during this time. Improving recurrent medical conditions that call for antibiotic use will improve treatment outcomes. Reducing local heat, sweat, and friction, as well as bowel disorders, is also important.

### Case history

A 36-year-old premenopausal woman presented with a 2-year history of urinary frequency, dysuria, and minor stress urinary incontinence. Urine culture, urodynamic studies, and cystoscopy were all normal. Additional medical history obtained included a 3-year history of chronic sinusitis, managed with multiple courses of oral antibiotics leading to monthly episodes of (confirmed) vulvovaginal candidiasis, usually during the premenstrual phase, and inadequately managed with over-the-counter antifungals. The patient also had frequent episodes of acute candidiasis in her twenties.

**Figure 2:**
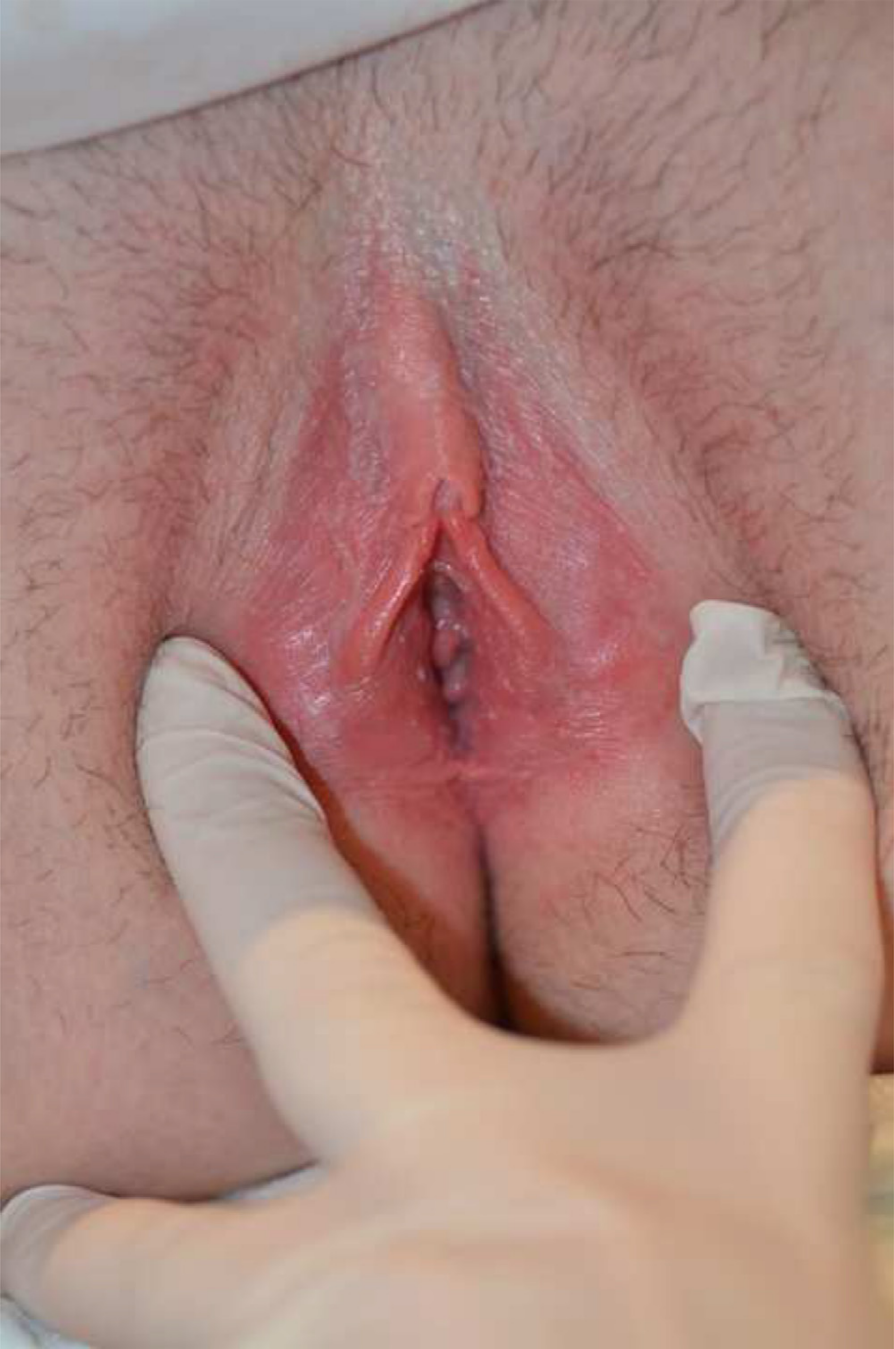

Examination showed a poorly demarcated, swollen, erythematous rash the involving labia minora and vaginal vestibule (Figure 2). The patient was given a provisional diagnosis of bladder dysfunction secondary to chronic vulvovaginal candidiasis.

Initial management included reducing heat, sweat, and friction, fluconazole 50 mg daily, and 1% hydrocortisone ointment twice daily for any vulval discomfort.

At 3 months of treatment, the patient was asymptomatic, bladder dysfunction was controlled, and examination was normal. Further management was per the CVVC guidelines (Gyan et al., 2013; Hong et al., 2014).

### Comment

This patient’s bladder dysfunction was secondary to chronic vulvovaginitis. Her condition was precipitated by long-term antibiotics. The history from her twenties is a clue to her susceptibility to CVVC. Treatment with an oral antifungal agent resolved her symptoms. The patient will need to take prophylactic antifungals when on antibiotics in the future.

## Lichen sclerosus

LS is an uncommon skin disease with a predilection for the genital skin (Lee et al., 2015). Its prevalence is estimated at up to 3% in patients age >50 years. The etiology remains unknown; however, there is a well-documented association with autoimmune disease, particularly thyroiditis and vitiligo. LS is important because, if not treated aggressively, it may significantly scar and deform the vulva. Furthermore, there is a lifetime 5% risk of squamous cell carcinoma in untreated disease. Therefore, these patients require lifelong observation and preventative treatment.

The most common presenting symptom is severe itch. It is sometimes painful as a result of excoriation or fissuring. Dyspareunia is also very common. However, LS can be completely asymptomatic, discovered by chance by the patient or during routine pelvic examination.

If left untreated, the labia minora eventually become reabsorbed and the clitoris becomes entrapped and buried, revealing an overall atrophic, shiny, white vulva missing normal anatomy. It is very typical for the labia minora to fuse in the midline. This brittle fusion tears easily during intercourse. Eventually, the introitus may become significantly stenosed, with pooling of urine within the vagina, simulating urinary incontinence. The typical appearance is a well-defined, white, sclerotic plaque with an atrophic wrinkled surface and areas of purpura and erosion. However, there are many variations. Edema, telangiectasia, purpura, and fissures may also be seen.

The distribution is very variable. The classic figure-of-eight distribution encircling the vulva, perineum, and perianal skin is uncommon. Often, only isolated areas of the vulval or perianal skin are affected. LS can involve the introital skin, but not inside the hymen.

Fortunately, LS is very responsive to topical corticosteroids, which are safe even with long-term use. It has been demonstrated that continuous treatment with a topical corticosteroid potent enough to render the skin objectively normal will not only prevent further scarring, but greatly reduce the risk of subsequent malignancy (Lee et al., 2015).

Treatment should be individualized. The broad principle should be to initiate treatment with a potent corticosteroid, used daily until skin texture and color have returned to normal, and then titrate down to a level that maintains remission. LS in adults is a lifelong disease that is unlikely to remit, and treatment cessation almost always results in relapse. Patients must understand that treatment may be for life and must continue even when symptoms have been completely controlled. Because most patients with LS are menopausal, estrogen replacement may be required as well if there is symptomatic atrophy.

### Case history

A 62-year-old postmenopausal woman presented with 4 years of dry vaginal dyspareunia. Vaginal estrogen was ineffective. In addition, pruritis ani was noted, for which hemorrhoid preparations was ineffective.

**Figure 3:**
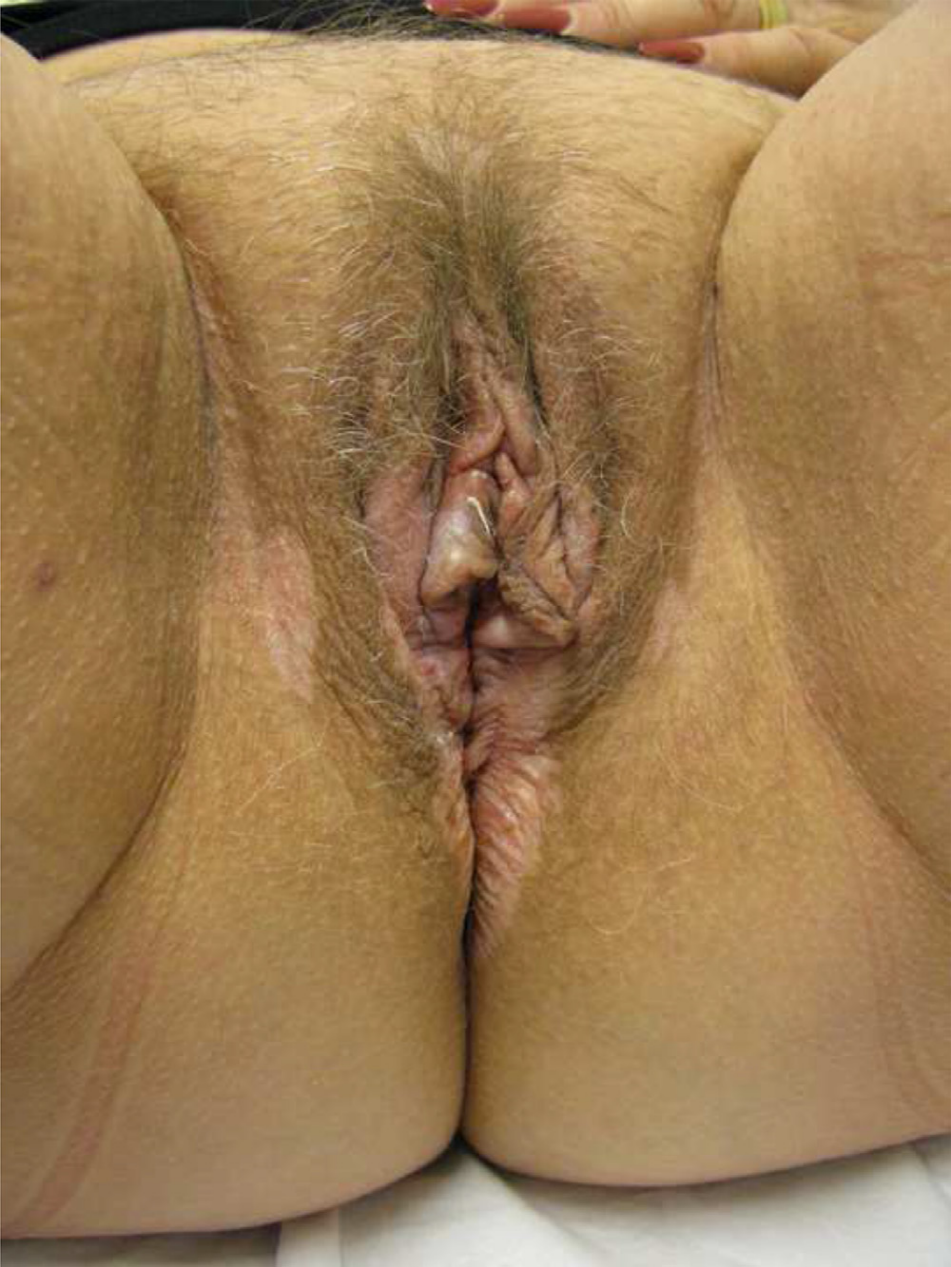

Examination showed a figure of eight of highly lichenified LS on vulval and perianal skin (Fig. 3). Biopsy confirmed the diagnosis. The patient was diagnosed with vulval and perianal LS.

Initial management included betamethasone dipropionate 0.05% in a modified vehicle, applied nocte. At 6 weeks of treatment, dyspareunia was improving, the pruritis ani was eliminated, and examination showed complete visual suppression of LS.

Further management consisted of a corticosteroid suppression regimen reduced to methylprednisolone aceponate ointment Monday through Friday and betamethasone dipropionate ointment Saturday and Sunday. At 3 months, dyspareunia was minimal but dry, and examination showed continuing visual suppression of LS and menopausal vulvovaginal atrophy.

The current LS management was continued, but vaginal estrogen was added. At 6 months, dyspareunia was eliminated, there was no pruritis ani, and examination showed continued visual suppression of LS.

### Comment

LS is easily missed. Although this postmenopausal patient eventually benefitted from topical estrogen, this treatment alone was inadequate. The biopsy-proven diagnosis of LS allowed for counseling to reassure the patient that topical corticosteroid is a safe, effective, long-term solution. Once the LS was treated, the addition of topical estrogen became effective. Prior to that, dyspareunia was related to pain from fissures and erosions. Future management with maintenance topical corticosteroid and estrogen will allow the patient to live a normal life.

## Summary

Disease and dysfunction of the pelvic viscera are intimately associated, and the bladder and urethra are more likely to be influenced by the more posterior viscera. This influence is largely via the all-encompassing pelvic floor complex. There is an additional influence on all pelvic viscera by spinal and hip disorders. Genital dermatoses are a common but underrecognized cause of bladder and urethral dysfunction.
